# Percutaneous Endovascular Coil Embolization of a Post‐Cholecystectomy Aberrant Right Hepatic Artery Pseudoaneurysm: A Case Report

**DOI:** 10.1002/ccr3.73318

**Published:** 2026-08-17

**Authors:** Shahan Ahmed, Faizan Ahmed, Ehsan Masud Kiani, Mahnoor Ahmed, Anis Ahmed, Syeda Qumreen Ahmad, Fatima Nazir, Kamil Ahmad Kamil

**Affiliations:** ^1^ Rawal Institute of Health Sciences Islamabad Pakistan; ^2^ Faisalabad Medical University Faisalabad Pakistan; ^3^ Ripha International University Rawalpindi Pakistan; ^4^ Rawalpindi Medical University Rawalpindi Pakistan; ^5^ Department of Surgery Benazir Bhutto Hospital Rawalpindi Pakistan; ^6^ Department of Internal Medicine Nasrat Bashir Curative Hospital Kandahar Afghanistan

**Keywords:** endovascular coil embolization, gall stones, hepatic artery pseudoaneurysm, post‐cholecystectomy vascular complications, pseudoaneurysm

## Abstract

Hepatic artery pseudoaneurysm (HAPA) is a rare but serious condition with significant mortality and morbidity. HAPA is a complication that can occur after cholecystectomy, particularly in patients who have variant hepatic arterial anatomy. We present a case of a 65‐year‐old male who developed hemobilia, right upper quadrant pain, and obstructive jaundice after open cholecystectomy. CT angiogram revealed a pseudoaneurysm of an aberrant right hepatic artery, which arose from the celiac trunk. The patient was successfully treated with percutaneous endovascular coil embolization which resulted in immediate hemostasis and resolution of his symptoms. Early recognition with imaging and a minimally invasive approach with urgent intervention is important for a successful outcome.

**Key Clinical Message**

Hepatic artery pseudoaneurysm is a rare but serious complication of cholecystectomy. It should be suspected in patients with unexplained upper GI bleeding, hemobilia, right upper quadrant pain, or jaundice. CT angiography enables early detection. Endovascular coil embolization is the preferred, safe, and effective treatment, supported by coordinated multidisciplinary care.

## 1 Introduction

**FIGURE 1:**
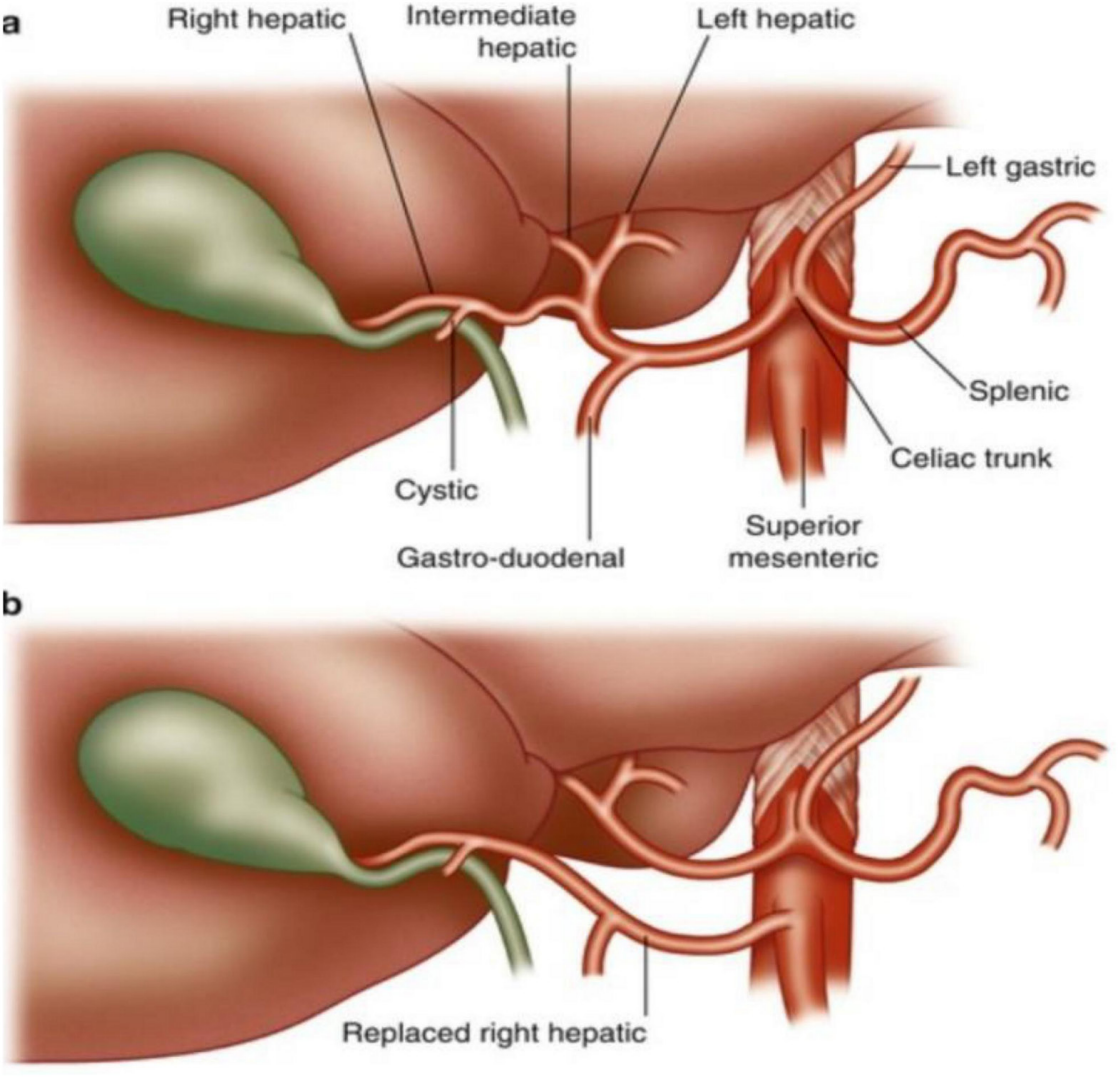
Normal anatomy of hepato‐biliary vasculature (a) and variant anatomy with aberrant RHA originating from the celiac trunk (b).

Vascular complications after cholecystectomy are rare but serious [1]. Variability in the surgical anatomy of the hepatic arteries increases the potential risk of injury during hepatobiliary surgery. Normally, the hepatic artery proper bifurcates into left and right hepatic arteries (RHA). But, in one third of cases, an aberrant right hepatic artery (aRHA) may originate from the superior mesenteric, gastroduodenal, right gastric artery, or the coeliac trunk [2] (Figure 1). In some cases, the RHA is an accessory, meaning an additional RHA is present alongside the normal one. Intraoperative misidentification of these variants increases the risk of trauma from surgical clips and electrocautery or due to infection after bile leak; all of which can damage the RHA with subsequent formation of a pseudoaneurysm [3].

The timing of presentation of pseudoaneurysms can vary from days to more than a year after surgery. Hemobilia is the most common presentation, with upper GI bleeding (90%), abdominal pain (70%), and jaundice (60%). Quincke's triad of melena, jaundice, and pain in the right upper quadrant occurs in 20%–40% of patients [4]. Rupture may present with intraperitoneal hemorrhage and carries a mortality rate as high as 50% [5]. Diagnosis is established with imaging; ultrasound demonstrates a pulsatile hypoechoic lesion [6], while contrast‐enhanced CT and angiography can confirm the pseudoaneurysm. Transarterial embolization is the preferred treatment method with a success rate of 85%; however, it may recur, requiring repeat procedures or surgery [7, 8]. Coils are often preferred for embolization, and results are mostly similar regardless of material. It is necessary to be cautious of incidental anatomical variations of cystic and hepatic arteries [9]. We present a challenging case of a right hepatic artery pseudoaneurysm that formed as a complication of cholecystectomy due to the variant anatomy of the right hepatic artery.

## 2 Case Presentation

A 65‐year‐old gentleman diagnosed with hypertension presented with intermittent right hypochondrial pain of 5 months' duration, which usually lasted for hours and was associated with vomiting. There were no clinical or laboratory features suggestive of acute cholecystitis. Ultrasonography revealed cholelithiasis, and a diagnosis of symptomatic gallstone disease was made. His preoperative work‐up, including a complete blood picture and liver function tests, was normal, and he was admitted to the hospital.

An open cholecystectomy was planned based on institutional practice and surgeon preference. Intraoperatively, Calot's triangle was identified, and the cystic artery and duct were clipped under vision and cut. While removing the gallbladder, massive bleeding was observed. Upon inspection, an aberrant right hepatic artery was found, which was ligated, while 02 pints of blood were transfused. Following hemostasis, a bile leak was noted, likely due to intraoperative biliary tract manipulation or minor ductal injury, and a T‐tube was placed with a plan for drainage and postoperative T‐tube cholangiogram.

Postoperatively, there was a gradual decrease in his T‐tube output. On the 10th postoperative day, he became apprehensive and agitated, and there was massive bleeding in the T‐tube drain and in the sub‐hepatic drain. He developed shock, for which he was resuscitated. His hemoglobin dropped to 5.6 g/dL, requiring blood transfusions. Over the next week, he became progressively jaundiced, and liver functions were consistent with an obstructive pattern with elevated bilirubin and cholestatic enzymes. The bleeding in the drains progressively decreased, the T‐tube drain output became clear, but jaundice and right upper quadrant pain worsened progressively over the next week.

### 2.1 Methods

The differential diagnosis of post‐cholecystectomy obstructive jaundice and right upper quadrant pain included a retained common bile duct stone, bile duct injury or ligation, bile leak or biloma, biliary stricture, sump syndrome (due to accumulation of debris in the distal CBD), cystic duct remnant syndrome, papillary stenosis, and sphincter of Oddi dysfunction. Other rare differential diagnoses included a hepatic artery pseudoaneurysm or portal vein thrombosis. The addition of hemobilia to the combination of obstructive jaundice and right upper quadrant pain significantly narrowed the differential diagnosis to a hepatic artery pseudoaneurysm. Though other rare differentials included portal injury, arteriobiliary fistula due to clip erosion or infection, bile duct injury with vascular communication, or non‐biliary, non‐vascular causes like peptic ulcer disease or malignancy (cholangiocarcinoma or ampullary carcinoma).

**FIGURE 2:**
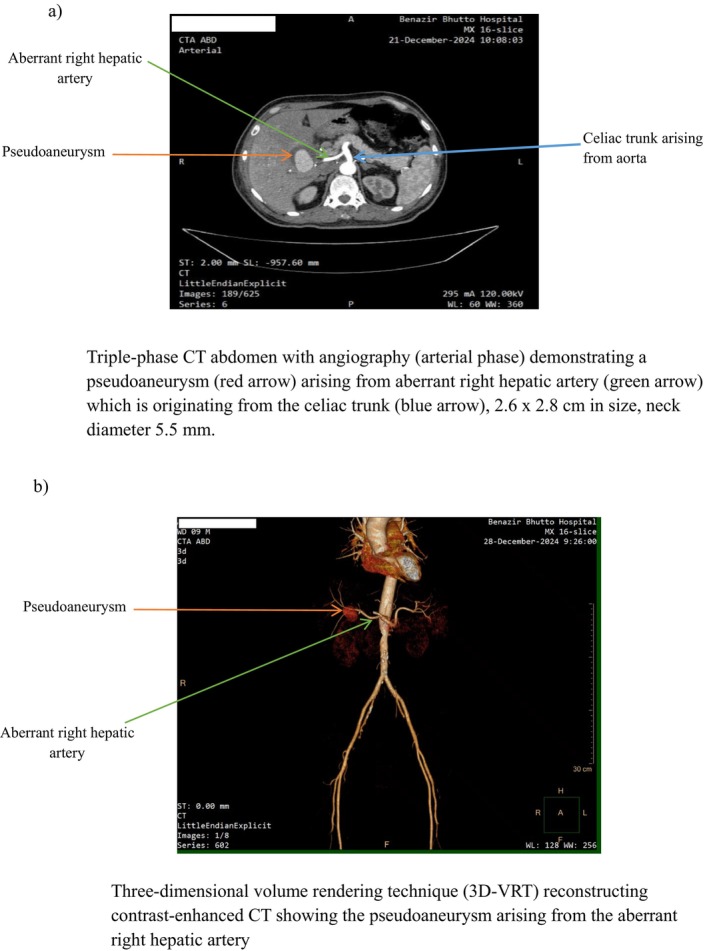
CT angiogram (a) and 3D volume‐rendered reconstruction (b) demonstrating the aberrant right hepatic artery pseudoaneurysm.

To find the cause, we performed a repeat ultrasound, which showed no intra‐abdominal collection; the T‐tube was in situ, but a pulsatile hypoechoic area at the porta hepatis was noted. Contrast‐enhanced CT (Figure 2a) showed a sacular aneurysmal dilatation 26.5 mm in diameter of the intrahepatic part of the right hepatic artery. Three‐dimensional volume rendering technique (3D‐VRT) reconstructed contrast‐enhanced angiogram (Figure 2b) confirmed that the aberrant right hepatic artery originated from the celiac trunk and the intrahepatic part was associated with a pseudoaneurysm.

**FIGURE 3:**
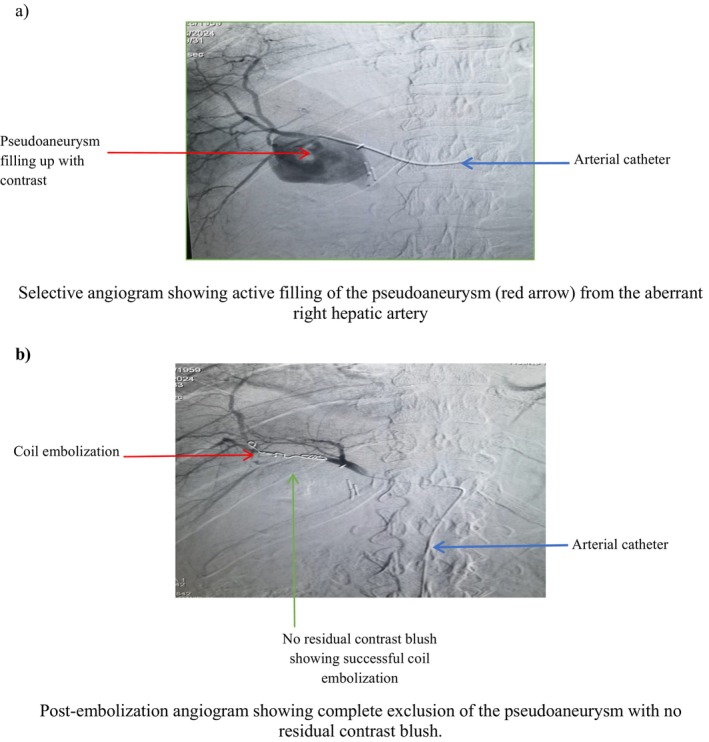
Pre (a) and post‐embolization (b) angiograms.

The case was discussed in a multi‐disciplinary meeting, and it was decided that the patient needed percutaneous trans‐arterial coil embolization of the pseudoaneurysm. The procedure was performed by the interventional radiologist through right femoral access with a 4 Fr C2 catheter. Wire was advanced into the distal right hepatic artery, and embolization was done with a metallic detachable coil of 0.035″ diameter. Coiling was done distal and proximal to the neck of the pseudoaneurysm (Figure 3a,b).

### 2.2 Conclusion and Results

Post‐embolization, the patient remained hemodynamically stable, but there were two minor episodes of bloody stools, which later subsided. Hemoglobin and liver functions showed an improving trend after the intervention. The T‐tube was removed after an unremarkable cholangiogram. The patient was discharged, and follow‐ups after 2 and 4 weeks showed progressive clinical improvement with no further episodes of bleeding. Post‐embolization angiogram at 2‐week follow‐up showed no filling of the pseudoaneurysm and filling of the distal right hepatic artery branches via collaterals (Figure 3b). At the 6‐month follow‐up, the patient has no active complaints and has demonstrated no complications.

## 3 Discussion

In our case, an intraoperative injury occurred to the aRHA that was originating from the celiac trunk, resulting in the formation of a pseudoaneurysm that bled into the biliary tree (hemobilia). Right hepatic artery pseudoaneurysm (rHAPA) is a rare, potentially life‐threatening vascular complication that can occur following hepatobiliary surgery, such as cholecystectomy. The formation of pseudoaneurysms following a cholecystectomy is rare, and the incidence has been reported at around 0.06%–0.6% [10]. When they do occur, the right hepatic artery is the most common vessel involved. In our situation, also unique, the RHA was aberrant, arising from the celiac trunk. Most pseudoaneurysms occur iatrogenically, most commonly from mechanical trauma (i.e., inadvertent laceration, aggressive clipping, traction injury), whereas thermal injury of the artery is less common [11].

These injuries typically present later; the pseudoaneurysm will often quietly balloon in size, and patients present days to weeks after surgery with nonspecific symptoms such as GI bleeding (hematemesis/melena), abdominal pain, or rarely jaundice [12]. In our case, the first bleed occurred approximately on the 10th postoperative day. The intermittent nature of the bleeding delayed diagnosis, emphasizing the difficulty of diagnosing HAPAs in the absence of high clinical suspicion.

Unmanaged HAPAs have a significant risk of rupture and exsanguinating hemorrhage, with mortality reported as high as 50%. Thus, timely diagnosis and urgent intervention are critical for good outcomes. The standard of care for a HAPA is endovascular embolization. Evidence shows that patients treated with transarterial coil embolization exhibit high technical success rates and lower morbidity than those treated with open surgical repair. Clinical practice guidelines from the Society for Vascular Surgery advocate for an endovascular‐first approach for visceral aneurysms, including HAPAs, with preservation of hepatic perfusion [13].

In this instance, the patient was promptly sent for angiographic embolization after the pseudoaneurysm was identified. A microcatheter was selectively advanced into the RHA branch feeding the pseudoaneurysm under fluoroscopic guidance, and the pseudoaneurysm was successfully occluded with detachable coils (0.035‐in. diameter). The coils were densely packed until cessation of blood flow into the pseudoaneurysm (a follow‐up angiogram showed obliteration of the pseudoaneurysm with no residual blush of contrast). The minimally invasive technique achieved immediate hemostasis, relieved obstructive jaundice, and avoided complications associated with an open procedure. This report also further emphasizes the importance of including vascular complications in the differential diagnosis of postoperative bleeding, especially with a delayed presentation. Access to relevant imaging with Doppler ultrasound and expedited CT angiography, if needed, provides early intervention preparations. An interdisciplinary approach between surgery and interventional radiology was required to facilitate the successful outcome. Advanced endovascular procedures, including coil embolization, can be performed effectively even in resource‐limited settings when the necessary expertise and infrastructure are available.

In conclusion, providers should have a high suspicion for hepatic artery pseudoaneurysm in patients who have undergone a cholecystectomy and present with an otherwise unexplained upper gastrointestinal bleeding, hemobilia, or obstructive jaundice, especially in patients with variant hepatic arterial anatomy. CT angiography followed by endovascular coil embolization is an effective minimally invasive treatment option and has lower morbidity than open surgical treatments. Awareness of this rare complication contributes to the development of clinical guidelines and helps optimize postoperative care.

## Author Contributions

**Shahan Ahmed:** conceptualization, methodology, writing – original draft, writing – review and editing. **Faizan Ahmed:** data curation, investigation, writing – original draft, writing – review and editing. **Ehsan Masud Kiani:** data curation, investigation, validation, writing – original draft, writing – review and editing. **Mahnoor Ahmed:** investigation, validation, writing – review and editing. **Anis Ahmed:** project administration, supervision, writing – original draft. **Syeda Qumreen Ahmad:** investigation, writing – original draft, writing – review and editing. **Fatima Nazir:** writing – original draft, writing – review and editing. **Kamil Ahmad Kamil:** writing – original draft, writing – review and editing.

## Funding

The authors have nothing to report.

## Ethics Statement

This case report was conducted in accordance with the Declaration of Helsinki. Ethical approval was obtained from the Institution.

## Consent

Written informed consent was obtained from the patient for publication.

## Conflicts of Interest

The authors declare no conflicts of interest.

## Data Availability

All available data can be made available on request by the journal for review.
